# Progress of Surgery

**Published:** 1874-07

**Authors:** Jno. E. Owens

**Affiliations:** Lecturer on Surgery, Rush Medical College, Chicago


ARTICLE III.—*Progress of Surgery.* By JNO. E. OWENS, M.D., Lecturer on Surgery, Rush Medical College, Chicago.

1. Tractile Method of Reducing Strangulated Hernia. By DANIEL LEASURE, M.D., of Pittsburg, Pa., (*American Journal of the Medical Sciences*, April, 1874.)

2. On Suspension in the Treatment of Fractures of the Leg. By JNO. H. PACKARD, M.D., one of the Surgeons to the Episcopal Hospital, Philadelphia. (*American Journal of the Medical Sciences*, April, 1874.)*

3. The Influence of Extension on the Intra-Articular Pressure and on the Articular Ends of Bones. By Dr. REYHER, of Dorpat. (*American Journal of the Medical Sciences*, April, 1874; *Medical Times and Gazette*, Jan. 31, 1874; and *Deutsche Zeitschrift fur Chirurg.*, band iv., heft 1.)

4. Traumatic Aneurism of the Femoral Artery by Injection of Ergotin. Recorded by Dr. PLAGGE, of Darmstadt. (*American Journal of the Medical Sciences*, April, 1874; *London Med. Record*, Feb. 11, 1874; *Betz's Memorabilien*, vol. xviii., part 1.)

5. Popliteal Aneurism Treated by the Introduction of Horse Hair into the Sac. By Dr. BRYANT, Guy's Hospital. (*American Jour. of the Med. Sciences*, April, 1874; *London Med. Record*, Feb. 11, 1874; *Guy's Hospital Gazette*, Dec. 6.)

6. Pyæmia in Private Practice. By PRESCOTT HEWETT, F.R.C.S. (*London Lancet*, April, 1874.)

7. Hypogastric Lithotomy. By SAMPSON GAMGEE, F.R.S.E. (*London Lancet*, April, 1874.)

8. A Series of Cases of Compound Fractures Successfully Treated on Conservative Principles at St. Bartholomew's Hospital. By Mr. CALLENDER. (*London Lancet*, April, 1874.)

9. Turpentine in Pyæmia. By Dr. J. SINCLAIR HOLDEN. (*London Lancet*, April, 1874.)

1. In this article five cases are cited in illustration of the reduction of strangulated hernia by "The Tractile Method." The first case occurred in 1843. In lifting a tub of wet clothing, the patient, a woman, forty years old, felt the protrusion (femoral)

* At the County Hospital, Dr. Powell has been employing this method with great satisfaction, in fractures of the thigh and compound fractures of the leg.

take place. Immediately pain, nausea and vomiting supervened; the former grew constant and intense, and the latter stercoraceous. After persistent but futile efforts at reduction, the patient being under the influence of anodynes and sedatives, anæsthetics not being then in use, it was decided to prepare to operate. Night came on in the meantime, and the patient's suffering grew more intense. Dr. Leasure, made desperate by her cries, and hardly knowing what more to try, stated to her husband that if he would suspend her by the limbs with her head downwards, he would make another attempt at reduction. "So turning his back to the bed, he sunk suddenly down, and placing one of the patient's legs over each shoulder as far as the hams, he seized an ankle in each hand, and drew her heels against his breast, then raising himself into the erect position the patient hung down along his back until only her head and shoulders rested on the bed." The hernial protrusion suddenly slipped away from touch into the abdominal cavity.

In the fall of 1853, Dr. Leasure met with a case of strangulated scrotal hernia, which defied his best efforts at reduction during many hours, having used anodynes, cold, heat, and prolonged compression and manipulation, whilst the patient was thoroughly under the influence of chloroform. Preparations for an operation were completed, but before making the incision it was decided to make a similar experiment to that so successfully performed ten years before. "Requesting an assistant to 'hunker down' by the bedside, the patient's legs were placed over his shoulders, and seizing an ankle in each hand, he drew them towards his breast, and rising to his feet, he carried the lower extremities up with him, until only his head and shoulders touched the bed." "Having pressed with both my hands," says Dr. Leasure, "down along the sides and front of the abdominal walls, as though trying to push the contents towards the diaphragm, the tumor was grasped by the right hand, the fingers passing well over the posterior part, and a slight effort made to draw the neck away from the belt of the stricture, when, without having made any pressure, I felt the posterior part of the tumor give a little slip, then another and, finally, with a rush the whole passed into the cavity."

Dr. Leasure has successfully employed this "tractile method" in the reduction of other cases of strangulated hernia after the best directed taxis had failed. The protruded viscera are, by this method, replaced by the force exerted from within by "traction" of the mesentery, the intestines and the omentum upon the hernial protrusion, and not to the "pushing in" process of ordinary taxis. Of course, the same general principles that apply to the treatment of hernia by taxis, should be rigidly observed in its treatment by the "tractile method." An irregular practitioner became aware of the efficiency of Dr. Leasure's method, and the latter cites a case in which the former, by a violent trotting of a patient in the "tractile position," tore the ileum and omentum from the imprisoned and gangrenous portion, and killed the patient.

2. This is an "old dish under a new cover," but Dr. Packard has done a good thing, not only in reminding surgeons of Dr. Nathan R. Smith's treatment of fractures by suspension, but also in setting forth the principles upon which it should be employed, and the advantages which it possesses.

In the article Dr. Packard claims for the treatment the following advantages:

(*a.*) "As to the efficiency of the treatment. The patient cannot possibly be kept altogether motionless, and if the limb is duly adjusted and properly swung, the fragments are made to follow each the movements of the other, so that their relative position is unchanged, even in cases of restless children."

(*b.*) "As to the comfort of the patient. Even from the very first he can be allowed to sit up in bed, reading, writing, or attending to business, a most important point, especially to persons in the more cultivated classes, who find the enforced idleness of lying flat on the back intolerably irksome.

"Again, by very slight turning the patient can shift his weight, so as to alter the bearing points of his body, thus avoiding much weariness, and often preventing the formation of bed-sores.

"Again, the bed-pan can be readily used, enabling us to dispense with the 'fracture-bed,' which is particularly objectionable in private practice.

"Once more, the frequent change of angle, both at the knee and hip, obviates, in great measure, the stiffening of these joints, and the use of the limb is regained much sooner than if the extended posture is maintained throughout the period of consolidation."

(*c*.) "As to the comfort of the surgeon. The risk of displacement is really so much diminished by the proper suspension of the limb, that a case so treated needs a less constant and anxious attendance than if this plan is not adopted. To country practitioners in dealing with distant cases, or to city surgeons who have many other demands on their time, this is a matter of no small moment."

Dr. Packard's article is well worthy of perusal by those who are not familiar with the treatment of fractures of the leg by suspension.

3. When filled with fluid, says Dr. Reyher, the knee joint always assumes a flexed position, and the intra-articular tension is considerably raised by slowly extending the limb. The degree of flexion which is thus imposed upon the limb when the cavity of the knee joint is completely distended, depends chiefly upon another condition, namely, the degree of tension of the muscles immediately surrounding it. If all the .muscles around the knee joint are preserved, its greatest capacity coincides with an angle of thirty degrees. Bennet teaches that the greatest capacity is with the leg flexed at an angle of sixty degrees, which is true only when the joint is entirely free from surrounding muscles. The reason assigned is that the quadriceps extensor, by means of its tendon, presses the patilla against the condyles whenever the joint is flexed; and the same applies to the gastronemius in its relation to the portion of the capsule behind the joint. In the case of a normal knee joint, a weight of forty pounds is required to separate the articular ends of the bones by direct extension.

The intra-articular tension of the knee joint is, generally speaking, diminished by extension; and that diminution becomes more appreciable the smaller the amount of fluid in the capsule and the greater the relaxation of the surrounding muscles. On the contrary, when the joint is quite full and the muscles are tense, the very slightest extension will considerably raise the intra-articular tension.

The following conclusions are arrived at by Dr. Reyher, viz.: It is possible to separate the ends of the bones at once by means of extension—that is, to produce "distraction"* of the joint;

* The complete mutual depuration of the opposed articular cartilages.

but to accomplish this, a weight of least thirty pounds is required, and this weight has probably never yet been applied to the knee joint of a living man. A smaller weight, for a longer time, may produce the same effect by a gradual extension of the ligaments. In cases where the joint contains much fluid—that is, in cases of abundant serous or purulent exudation—the intra-articular tension rises very high, and forcible extension would sooner produce a rupture of the capsule than "distraction" of the joint. Any attempt at extension in such cases would be both useless and dangerous. But it might, nevertheless, be important in such cases to separate the articular ends of the bones, in order to prevent all pressure and the ulceration of the cartilages, which might result. The author accordingly proposes to tap the joint subcutaneously first, and then try "distraction."

(The use of the aspirator would doubtless be an improvement upon ordinary tapping in such emergencies).

Dr. Albert, of Vienna, has formed a very similar opinion of the value of extension in the treatment of inflamed joints. After a great number of similar experiments in all joints, he has found that where they are fully distended, the intra-articular pressure always rises by extension; and that, in the case of the hip joint, especially, "distraction" is quite impossible.

4. A man was accidently stabbed with a small bread knife, about two fingers' breadth, just beneath Poupart's ligament, and the result was a traumatic aneurism. An ice-bag was applied, and digitalis and morphia administered internally.

The tumor increased, and two days afterwards a solution containing 2½ parts of ext. of ergot, and 7½ each of spirits of wine and glycerine, was subcutaneously injected on the inner side of the tumor, and the ice-bag assiduously applied. The aneurismal thrill lessened on the following day. The injections were continued during the next three weeks. At the end of that period there was visible diminution of the impulse, swelling, and sensibility to pressure. The patient could bear slight pressure for an hour or two at a time, thrice daily, and the ergot injection was discontinued. A fortnight later the impulse was scarcely perceptible, the tumor being scarcely larger than a hen's egg and not very sensitive. Rosar's tourniquet was applied, and the man was sent home. A month later he presented himself with the aneurism cured.

5. The aneurism was of some standing, its presence having been recognized six or seven months previously by the patient, who suffered from initial disease of the heart, and undoubtedly had very bad arteries. Pressure, productive of some hardening of the aneurism, had been tried till the patient was unable any longer to bear it. Epistaxis supervened, due doubtless to the initial regurgitation. The treatment by ergot was tried, as recommended by Langenbeck, but although it improved the pulse and stopped the epistaxis, it had no effect upon the aneurism, which was evidently increasing in size. Mr. Bryant introduced a very fine trocar and canula, and, withdrawing the former, he passed through the canula about eighteen feet of horse hair. This was accomplished in a few minutes, three threads of hair being pushed in together. Blood flowed through the canula, though the artery was compressed till the end of the operation, when it ceased; and when the canula was withdrawn, there was no bleeding from the wound. The leg having been covered with cotton wool, the patient was removed to the ward, the aneurism still pulsating, but not so strongly.

The patient had since died.

Dr. Levis had treated a case by this method in the Pennsylvania Hospital. The result was likewise unfortunate.

6. In an address before the Clinical Society of London, the author cites the history of twenty-three cases of pyæmia occurring in private practice. In three of the cases the disease grew out of gonorrhœa. An operation was performed in only six instances. The operation in four cases was of a trifling nature, namely, a single thread seaton, the removal of a small wart on the heel, one from the scrotum, a small sebaceous tumor of the scalp, and two cases of amputation of the breast. "The first four cases were all in different years, and not in the same locality. The last two were in the same year, and within a month of each other; one was in town, and the other in the country." The third case is mentioned among those of recovery, the patient having had two attacks of pyæmia at several years' interval, and in different localities. "Of the remaining seventeen cases, in which no operation had been performed, there was a broken surface in eleven, and in six there was not even an abrasion." "Of the eleven in which there was a broken surface, it was but small in ten; ulceration of small sero-cystic tumor of the breast, of abscess in two, of tonsils in two, of bowel in typhoid fever in three, a needle broken in the leg, a small splinter of wood in the great toe. The eleventh case was a compound dislocation of the elbow. The six cases, in which there was no abrasion, were, slight injury of the foot, followed by suppuration, inflammation of the lateral sinus of the internal jugular vein, in connection with discharge from the ear after measles, etc. Of these seventeen cases, none occurred at the same period or in the same locality. As to the locality of the twenty-three cases, sixteen occurred in town and seven in the country. Of the sixteen in town, all, with one exception—that of a young girl, who, after running a needle into her leg, was admitted with pyæmia into the hospital—were in the best parts of the town, scattered about in good houses of good sizes, with well ventilated bed rooms, well cared for, and, in fact, to all appearances, under the most favorable conditions. The seven country cases were in different parts, and widely separated from each other. Their conditions were excellent."

Dr. Hewett concludes that the causes of pyæmia are still to be worked out.

7. Dr. Gamgee, after expressing himself as averse to innovation and changes for the mere sake of novelty and variety, and after referring to the brilliant success of labial lithotomy in the hands of his countrymen, concurs in the opinion that supra-pubic lithotomy has advantages in exceptional cases. In illustration of the latter he reports the following case:

The patient, a pale, puny little girl for her age, (eight years and nine months), had long suffered from stone in the bladder. The stone was large, occupied one position, and the sound could not be passed around it. The pelvic outlet was very narrow, which fact, together with the size and fixity of the stone, led the operator to select the supra-pubic method. "On opening the tiny, thickened bladder, it was nearly filled with the calculus, which could not be removed without peeling off from its posterior surface the adherent vesical mucous membrane."

"The stone weighed 305 grains, and measured $1\frac{5}{8}$ in. x $1\frac{1}{8}$ in. x $\frac{7}{8}$ in. The wounds in the bladder and abdominal wall were closed by separate metallic sutures. The external ones were cut short, and not removed till the eighth day; but the ends of the two sutures in the vesical wall were left long, lightly twisted, and brought through the outer wound, so that they could be easily untwisted and slipped through on the fourth day. A catheter was kept in the bladder for a week. The wounds healed directly, and the patient left the hospital on the fourteenth day."

8. It is stated that this series of cases may be taken as representing the average success which Mr. Callender has obtained for a long time past.

(*a.*) Compound fracture of the entire lower tract of the humerus, extending into the elbow-joint. The arm was placed on an angular splint, and swung. The wound was covered with carbolized oil on lint, and over this was laid a thick layer of cotton-wool. The patient was injured on May 3rd. On Nov. 20th he was sent to the Convalescent Hospital, some sinuses in the arm still remaining open. There was some movement at the elbow-joint, and the prospects for further improvement were good.

(*b.*) Compound fracture of the right astragalus, and comminuted fracture of the tibia and fibula, extending into the ankle and into some of the tarsal joints. The limb was supported by splints, and the wound systematically washed out with carbolic acid lotion, and covered with carbolized oil on lint and cotton-wool. Five weeks after admission the astragalus necrosed and was removed.

