# Errata

**Published:** 1874-09

**Authors:** 


***Errata.***

On page 417, July No. of the JOURNAL, in the foot-note, the word "depuration" should be *separation.*

On page 420, in the 18th line from the bottom, the word "labial" should be *lateral.*

